# Early-Stage *Staphylococcus aureus* Bloodstream Infection Causes Changes in the Concentrations of Lipoproteins and Acute-Phase Proteins and Is Associated with Low Antibody Titers against Bacterial Virulence Factors

**DOI:** 10.1128/mSystems.00632-19

**Published:** 2020-01-21

**Authors:** Stephan Michalik, Nandakumar Sundaramoorthy, Annette Murr, Maren Depke, Uwe Völker, Barbara M. Bröker, Hege Vangstein Aamot, Frank Schmidt

**Affiliations:** a Department Functional Genomics, Interfaculty Institute for Genetics and Functional Genomics, University Medicine Greifswald, Greifswald, Germany; b ZIK-FunGene, Department of Functional Genomics, Interfaculty Institute for Genetics and Functional Genomics, University Medicine Greifswald, Greifswald, Germany; c Institute of Immunology and Transfusion Medicine, Department of Immunology, University Medicine Greifswald, Greifswald, Germany; d Department of Microbiology and Infection Control, Akershus University Hospital, Lørenskog, Norway; Princeton University

**Keywords:** DIA mass spectrometry, *Staphylococcus aureus*, biomarker, bloodstream infections, immunoproteomics, proteomics

## Abstract

Systemic and quantitative investigations of human plasma proteins (proteomics) and Staphylococcus aureus-specific antibodies (immunoproteomics) provide complementary information and hold promise for the discovery of biomarkers in Staphylococcus aureus bloodstream infection (SABSI). Usually, data-dependent acquisition (DDA) is used for proteome analysis of serum or plasma, but data-independent acquisition (DIA) is more comprehensive and reproducible. In this prospective cohort study, we aimed to identify biomarkers associated with the early stages of SABSI using a serum DIA proteomic and immunoproteomic approach. Sera from 49 SABSI patients and 43 noninfected controls were analyzed. In total, 608 human serum proteins were identified with DIA. A total of 386 proteins could be quantified, of which 9 proteins, mainly belonging to acute-phase proteins, were significantly increased, while 7 high-density lipoproteins were lower in SABSI. In SABSI, total anti-S. aureus serum IgG was reduced compared with controls as shown by immunoproteomic quantification of IgG binding to 143 S. aureus antigens. IgG binding to 48 of these anti-S. aureus proteins was significantly lower in SABSI, while anti-Ecb IgG was the only one increased in SABSI. Serum IgG binding to autoinducing peptide MsrB, FadB, EsxA, Pbp2, FadB, SspB, or SodA was very low in SABSI. This marker panel discriminated early SABSI from controls with 95% sensitivity and 100% specificity according to random forest prediction. This holds promise for patient stratification according to their risk of S. aureus infection, underlines the protective function of the adaptive immune system, and encourages further efforts in the development of a vaccine against S. aureus.

**IMPORTANCE** S. aureus sepsis has a high complication and mortality rate. Given the limited therapeutic possibilities, effective prevention strategies, e.g., a vaccine, or the early identification of high-risk patients would be important but are not available. Our study showed an acute-phase response in patients with S. aureus bloodstream infection and evidence that lipoproteins are downregulated in plasma. Using immunoproteomics, stratification of patients appears to be achievable, since at the early stages of systemic S. aureus infection patients had low preexisting anti-S. aureus antibody levels. This strengthens the notion that a robust immune memory for S. aureus protects against infections with the pathogen.

## Abstract (precis)

S. aureus sepsis has a high complication and mortality rate. Given the limited therapeutic possibilities, effective prevention strategies, e.g., a vaccine, or the early identification of high-risk patients would be important but are not available. Our study showed an acute-phase response in patients with S. aureus bloodstream infection and evidence that lipoproteins are downregulated in plasma. Using immunoproteomics, stratification of patients appears to be achievable, since at the early stages of systemic S. aureus infection patients had low preexisting anti-S. aureus antibody levels. This strengthens the notion that a robust immune memory for S. aureus protects against infections with the pathogen.

## INTRODUCTION

Staphylococcus aureus is the second most common cause of bloodstream infections (BSI) largely due to its virulence potential and omnipresent occurrence as a colonizer (1). The 30-day case fatality rates are reported around 20%, and the mortality rates are estimated to be 2 to 10 deaths annually per 100,000 population (2). The clinical outcome of S. aureus bloodstream infections (SABSI) is dependent on a complex combination of several factors including bacterial characteristics (3), host innate and humoral immune responses (4, 5), and underlying diseases (3). However, the high mortality rate could also reflect insufficient laboratory diagnostics, as each hour of delay in diagnostics increases the mortality rate (6, 7), because delayed and suboptimal antibiotic therapy negatively affects the clinical outcome (8).

To date, no single laboratory test accurately diagnoses bloodstream infections (9). The majority of biomarkers lack sufficient sensitivity or specificity (7). At present, C-reactive protein (CRP) and procalcitonin (PCT) are commonly used as biomarkers for sepsis, but their diagnostic value in bloodstream infections is still controversial (10, 11). In the case of SABSI, it is likely that networks of numerous virulence genes are expressed by S. aureus bacteria in response to distinct host signals (12).

In recent years, proteomics have been used to identify biomarkers (7) mainly from liquid biopsy samples like blood, which is the most commonly used patient material in clinical diagnostics. However, the blood plasma proteome is very complex and covers a large dynamic range spanning approximately 9 orders of magnitude from most-abundant functional plasma proteins like albumin to least-abundant signal proteins like interleukin 6 (IL-6) (13).

Protein quantification via mass spectrometry (MS) can rely on different data acquisition strategies: data-dependent acquisition (DDA) and data-independent acquisition (DIA). The most common mass spectrometric method is DDA, where a defined number of precursor ions have their *m/z* values recorded in a survey scan (MS1 spectrum) followed by fragmentation of the most abundant ion precursors resulting in a tandem mass spectrometry (MS/MS) or MS2 spectrum. In contrast, DIA performs predefined MS/MS fragmentation and data collection regardless of sample content. The latter protein quantification is more sensitive and accurate than DDA (14). The power and reliability of the DIA method for proteome analysis under S. aureus infection conditions have recently been published (15). However, only a limited number of reports focusing on the application of proteomic analysis of clinical samples from BSI patients are available (16, 17).

How the host maintains equilibrium with S. aureus—or fails to do so—is a microbiological and immunological puzzle, since most individuals are exposed to these bacteria during their lifetime and the bacteria can change from harmless residents to life-threatening enemies within one individual host. Immunoproteomics permits the comprehensive analysis of the adaptive immune response by identifying and measuring antigenic proteins or peptides along with the adaptive immune response against them. In S. aureus research, suspension array technology (e.g., Luminex) has been used, allowing simultaneous quantification of up to 500 different bacterial antibody-binding proteins (18). Immunoproteomic studies have shown that titers of specific serum IgG antibodies at baseline are correlated with disease progression. Moreover, prospective studies demonstrated that a strong anti-S. aureus antibody response develops during SABSI (5, 19, 20).

In this prospective cohort study on SABSI patients, we aimed to identify biomarkers associated with the early stages of SABSI using a serum DIA proteomic and immunoproteomic approach.

## RESULTS

### Patients.

Forty-nine SABSI patient and 43 control patient samples were included. Clinical data are presented in Table 1. The median age for the SABSI patients was 67 years (range, 28 to 95 years), and 33/49 (67%) were male. Thirteen patients (27%) had diabetes, and 15 (31%) were nasal S. aureus carriers. The majority were diagnosed with sepsis (42/49, 86%), whereas no SABSI patient had septic shock. Of the 49 patients, 7 died within 30 days (14%). Two control patients died within 1 year of elective surgery (5%), of which the first death was not due to postoperative infection or BSI and the second death was 6 months after the last checkup at the hospital. Cause of death is therefore unknown. There were no differences in age, gender, diabetes status, S. aureus nasal carriage, or total serum IgG concentrations between the SABSI patients and the controls. P-albumin (plasma albumin) was lower and CRP was higher in SABSI patients than in the controls (Table 1).

**TABLE 1:** Demographic and clinical data on patients with Staphylococcus aureus bloodstream infections (SABSI) and control subjects

Characteristic	SABSI	Controls	*P* value
*n*	49	43	
Age (median, yr) (range)	67 (28–95)	68 (23–87)	0.854 (Mann-Whitney U test)
Male gender (no.)	33	27	0.647 (Pearson chi-square test)
Severity of SABSI (no.)			
Sepsis	42		
Severe sepsis	7		
Septic shock	0		
30-day all-cause case fatality (no.)	7		
Diabetes (no.)	13	6	0.137 (Pearson chi-square test)
S. aureus nasal carrier (no.)	15	12	0.776 (Pearson chi-square test)
Time point collection of sera (no.)			
Within 24 h	30		
Within 48 h	14		
Within 72 h	5		
P-albumin^*a*^ (median, g/liter, reference range of 36–45 g/liter) (range)	33 (20–44)	44 (33–54)	<0.001 (Mann-Whitney U test)
CRP (mg/liter, reference range of <5 mg/liter) (range)	195 (10–500)^*b*^	3 (3–31)	<0.001 (Mann-Whitney U test)
Total IgG (median, g/liter) (range)	9.9 (3.8–18.7)	9.9 (5.3–18.4)	0.959 (Mann-Whitney U test)

a Not tested in two patients and one control.

b Not tested in one patient.

### Clinical S. aureus isolates.

**FIG 1:**
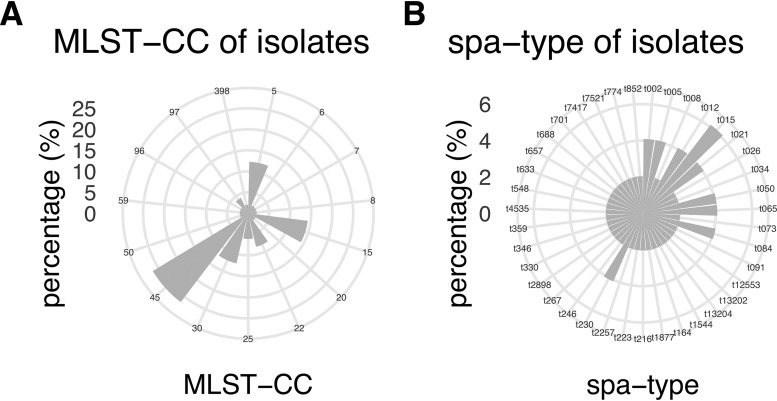
Radar plot displaying the multilocus sequence type (MLST) CC and *spa* type distribution of the 49 SABSI isolates.

The SABSI isolates clustered to 15 clonal complexes (CCs) with CC45 (27%), CC15 (14%), CC5 (12%), and CC30 (12%) being the most frequent (Fig. 1). Of the 39 SABSI *spa* types, the most frequent *spa* type was t015 (6%) (Fig. 1). S. aureus nasal carriage was identified in 31% of the SABSI patients and 28% of the controls (*P* = 0.776, Table 1). All but one SABSI patient (14/15, 93%) had isolates of the same CC in both BSI and nasal isolates, indicating endogenous infection. The most frequent of the 7 CCs in the controls was also CC45 (3/9). However, DNA microarray analysis was not performed on 3 isolates. Only *spa* type t350 (2/12) was identified in more than one control isolate. Two SABSI patients had methicillin-resistant S. aureus (MRSA), of which one also was positive for Panton-Valentine leukocidin (PVL). Two additional SABSI patients had methicillin-susceptible S. aureus (MSSA) positive for PVL. None of the controls had MRSA or were positive for PVL.

### DDA library construction and workflow for serum-specific DIA analysis.

In order to quantify proteins in sera from healthy and diseased humans, it is of importance to adjust the spectral DIA library to the sample origin. Therefore, DDA mass spectrometry (MS) data from around 90 measurements were considered. To extend the number of measurable peptides, we included plasma samples from different clinical cohorts and analyzed 20 samples of depleted plasma in addition to 70 samples of undepleted plasma. When applying a strict peptideProphet plus iProphet score of <0.3% false-discovery rate (FDR), 780 proteins were finally included in the human blood-specific protein DIA-MS library (see Table S1 in the supplemental material).

10.1128/mSystems.00632-19.2 TABLE S1 DIA-MS library. Download Table S1, TXT file, 23.3 MB. Copyright © 2020 Michalik et al. 2020 Michalik et al. This content is distributed under the terms of the Creative Commons Attribution 4.0 International license.

**FIG 2:**
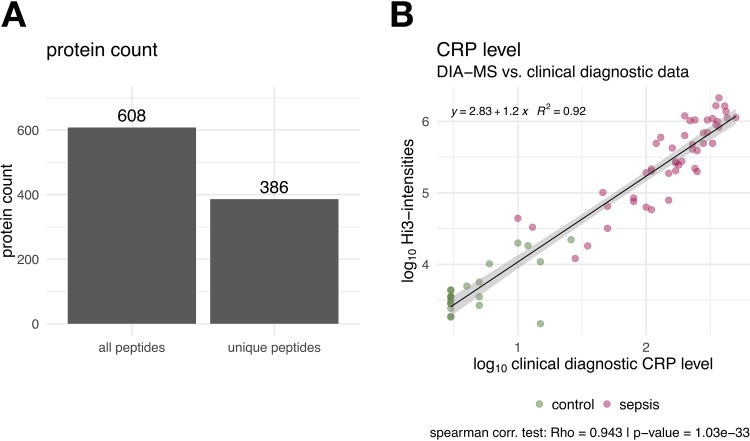
(A) Identification of serum proteins from both patient and control subjects. In total, 608 proteins were identified in all samples by DIA-MS. For further Hi3 (top 3 peptide quantification) quantitation, only protein hits with at least three unique peptides were considered. (B) Correlation of C-reactive protein (CRP; mg/liter) concentrations measured by ELISA and DIA-MS (Hi3 intensities). Control patient values are colored in green, and values of patients with S. aureus bloodstream infection are in red.

For serum proteome analysis, we used <1 μl of nondepleted serum, which was treated by tryptic digestion and measured by Q Exactive DIA. We did not deplete the most abundant proteins to avoid uncontrolled removal of interacting proteins, as this could interfere with precise quantification. Proteins were identified and quantified in 43 sera from control individuals and all 49 sera from people with SABSI using the Spectronaut analysis pipeline (21) containing the human serum-specific spectral library (780 proteins). In total, 608 proteins were identified. Using only unique peptide identifications, 386 proteins were used for quantitation via the Hi3 method (Fig. 2). This workflow increased the protein coverage of nondepleted human serum/plasma by a factor of 2 compared to DDA measurements (Spectronaut raw data are in Table S2).

10.1128/mSystems.00632-19.3 TABLE S2 DDA measurements, Spectronaut raw data. Download Table S2, XLSX file, 96.6 MB. Copyright © 2020 Michalik et al. 2020 Michalik et al. This content is distributed under the terms of the Creative Commons Attribution 4.0 International license.

To validate the DIA-MS method, we tested the correlation of the DIA-MS data with the enzyme-linked immunosorbent assay (ELISA) measurements of C-reactive protein (CRP), the gold standard in clinical research (Fig. 2A). The two methods show excellent correlation as shown in Fig. 2B (*R*^2^ = 0.92).

### DIA-MS serum protein profiles in serum samples from healthy and septic individuals.

**FIG 3:**
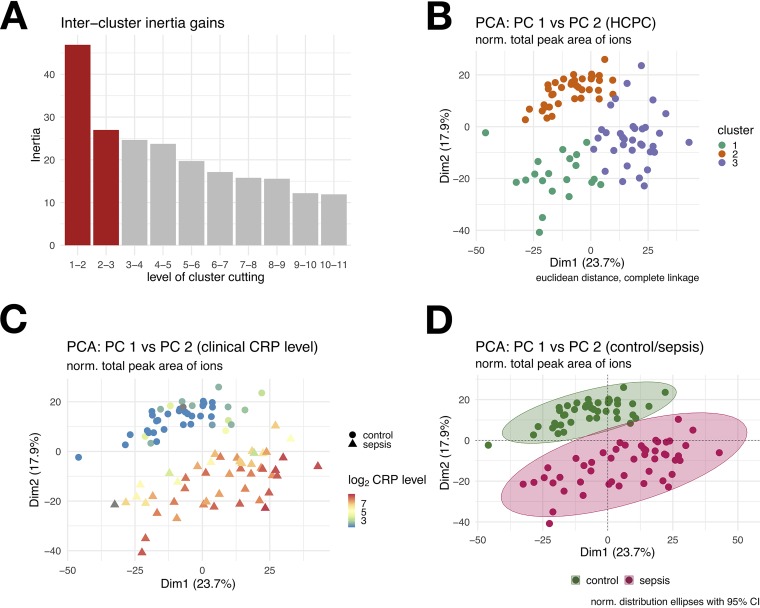
(A) Bar plot of inertia gains of agglomerative hierarchical clustering on PCA results. Red bars versus gray bars depict the cluster tree cut level. (B) PCA plot of DIA intensities where the points are colored by agglomerative hierarchical clustering on PCA results (3 clusters). (C) PCA plot of DIA-MS intensities where the points are colored by their corresponding log_2_ CRP level (gray indicates undetermined) and the point shape indicates the patient group (sepsis or control group). (D) PCA plot of DIA-MS intensities in controls (green) and in patients with S. aureus bloodstream infection (red).

Serum samples were obtained early during the disease course, no later than 4 days after symptom onset in all but 19 patients (39%). As expected, the CRP values of SABSI samples were significantly higher than those of the controls (Fig. 2B). To compare serum samples of control and SABSI patients, the data were normalized (Fig. 3A) and subjected to a principal-component analysis (PCA) (Fig. 3B). Strikingly, the PCA clearly separated SABSI patients from controls and showed no overlap (Fig. 3C).

**FIG 4:**
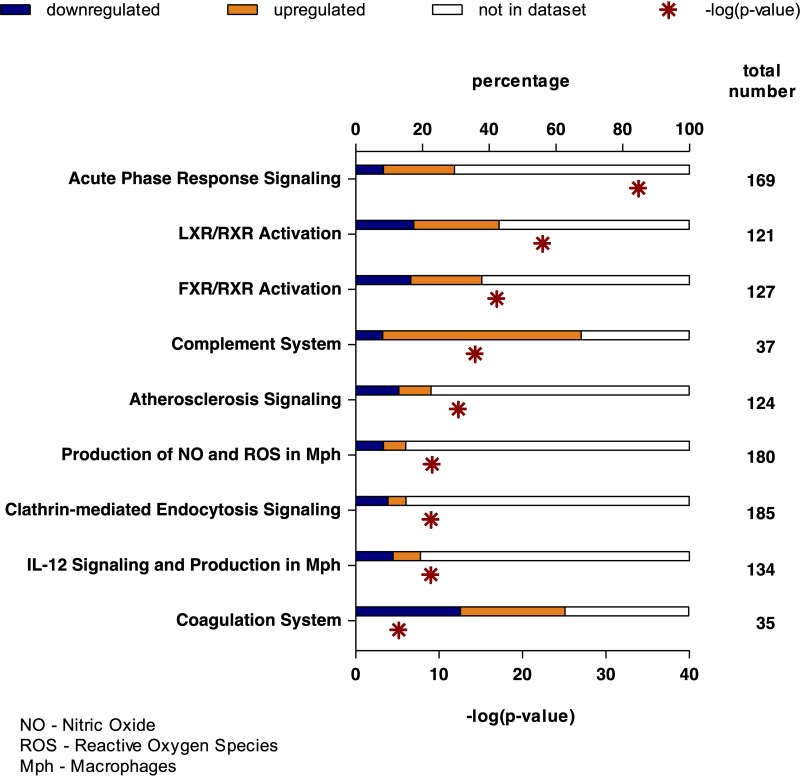
Serum proteins and related functional pathways significantly up- or downregulated in patients with S. aureus bloodstream infection compared to controls. IPA (Qiagen Bioinformatics, Redwood City, CA) of proteins with significant differences between the two groups (*P* < 0.05, FC > 1.5).

Considering only significant differences between the groups (*P* < 0.05, fold change [FC] > 1.5), the nine pathways most significantly affected during SABSI were identified by the program Ingenuity Pathway Analysis (IPA, Fig. 4). Most of the proteins belonging to the acute-phase response signaling pathway were highly upregulated in patients suffering from SABSI as well as proteins related to the liver X/retinoid X receptor (LXR/RXR) and farnesoid X receptor/retinoid X receptor (FXR/RXR) activation and the complement system (Fig. 4). Selected proteins with significant alteration between the two groups are shown in Table 2. Relative abundances of serum proteins like CRP, haptoglobin (HPT), α1-antitrypsin (A1AT or AAT), α1-acid glycoprotein 1 (A1AG1), von Willebrand factor (VWF), CD14, serum amyloid A1 (SAA1), and SAA2 were at least 1.9-fold higher in subjects with SABSI than in controls (Table 2). Vice versa, proteins mainly belonging to the apolipoprotein family were significantly lower in SABSI and are part of the high-density lipoprotein (HDL) lipid metabolism.

**TABLE 2:** Selected proteins (more than two unique peptides) with significant alterations in S. aureus bloodstream infections

Function (Ingenuity Pathway Analysis)	Protein abbreviation	Protein name	Regulation factor
Host defense	CRP	C-reactive protein	45.9
Antioxidant defense	CERU	Ceruloplasmin	1.5
Acute-phase response	HPT	Haptoglobin	3.0
	A1AG1	α1-Acid glycoprotein 1	2.4
	SAA1	Serum amyloid A1	99.3
	SAA2	Serum amyloid A2	60.1
Protection from proteolysis	A1AT	α1-Antitrypsin	1.9
Promoting adhesion of platelets	VWF	von Willebrand factor	3.3
Coreceptor for bacterial lipopolysaccharide	CD14	Cluster of differentiation 14	2.1
Lipoprotein metabolism	APOA1	Apolipoprotein A1	−1.5
	APOA2	Apolipoprotein A2	−2.1
	APOB	Apolipoprotein B	−1.2
	APOC1	Apolipoprotein C1	−2.1
	APOC2	Apolipoprotein C2	−2.2
	APOM	Apolipoprotein M	−1.4
Serine protease inhibitor	KAIN	Kallistatin	−1.9

### Quantification of S. aureus-specific IgG antibodies.

The total IgG serum concentrations were determined by turbidimetry. The median concentrations were 9.9 g/liter in both SABSI patients and controls (Fig. S1). A similar, equal proportion for detected IgG subtypes 2 and 3 was observed in the control versus SABSI in the DIA-MS experiments (see the supplemental material).

10.1128/mSystems.00632-19.1 FIG S1 Box plots of the total IgG concentrations in control and sepsis subjects measured by turbidimetry. Both groups show a median serum concentration of approximately 10 g/liter. Download FIG S1, TIF file, 1.0 MB. Copyright © 2020 Michalik et al. 2020 Michalik et al. This content is distributed under the terms of the Creative Commons Attribution 4.0 International license.

Next, specific IgG binding to 143 S. aureus proteins and autoinducing peptides (AIPs) was measured using the Flexmap 3D system (see list of S. aureus proteins in the supplemental material). The median relative IgG response intensity spanned more than 6 orders of magnitude, ranging from 1.01e+01 (weakest binding of antigen SACOL1802) to 2.58e+07 (strongest binding of antigen SAOUHSC_02161). Besides interindividual variation in the strength of the humoral immune response to S. aureus, this reflects significant differences in immunogenicity between the tested S. aureus antigens. Most highly immunogenic antigens belong to extracellular proteins or those associated with the bacterial cell wall. But there are also exceptions: the enzymes PurA, Tig, Tuf, and CitC and the chaperone GroEL have no signal sequences and are intracellular factors according to PSORTb 3 (22) or SignalP 5.0 (23), as well as according to analyses of subproteomes of S. aureus (24). Nevertheless, IgG binding to them was unexpectedly high and still in the lower middle range.

**FIG 5:**
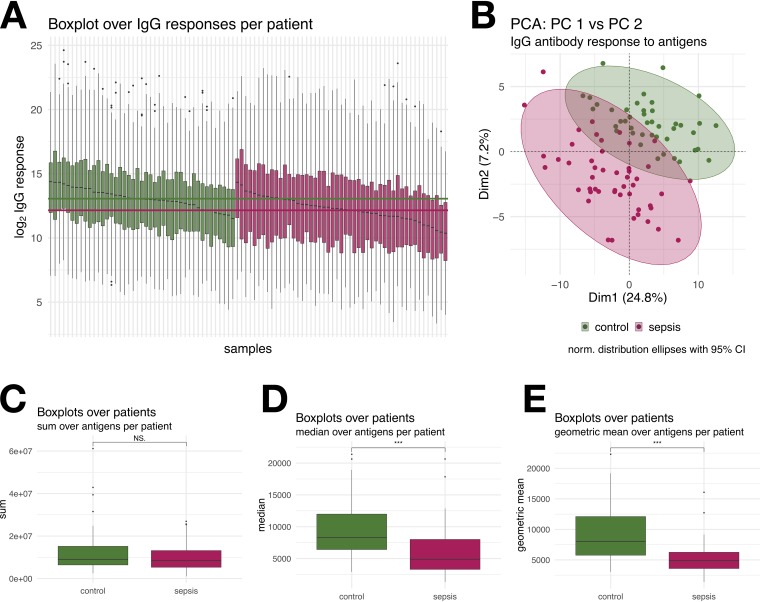
(A) Box plot of 143 S. aureus-specific IgG signals (mean fluorescence intensity) depicted for each of the 43 control (green) and 49 sepsis (red) subjects. Horizontal lines in green and red indicate the median values of control and sepsis subjects, respectively. (B) Principal-component analysis (PCA) of antibody responses against 143 candidate S. aureus antigens in control (green) and sepsis (red) patients. (C to E) Box plots of sum (C), median (D), or geometric mean (E) of S. aureus-specific IgG signals (mean fluorescence intensity) over all antigens per patient. Groups were statistically compared using a two-sided Wilcoxon test.

To estimate the total individual IgG response to S. aureus, first the intensities of IgG binding to all 143 antigens were summed for each patient and control individual. In previous studies, this number correlated well with serum IgG binding to total S. aureus extracellular protein extracts as measured by immunoblotting (5). SABSI patients had lower anti-S. aureus IgG levels than the controls, but the difference did not reach the significance threshold. However, the species S. aureus is very heterogeneous in the genomic sequence of the different strains. Investigating the S. aureus pangenome, 19% of the genes belong to the core, 39% belong to the accessory genome, and 42% are unique (25). The antigen panel used for the IgG response detection contains both conserved and variable proteins of S. aureus strains. Thus, subjects may not have been exposed to all antigens in the panel. To account for this, we compared the median and geometric mean of IgG binding over all antigens per patient, both of which were significantly lower in SABSI than in controls (Fig. 5B and C).

**FIG 6:**
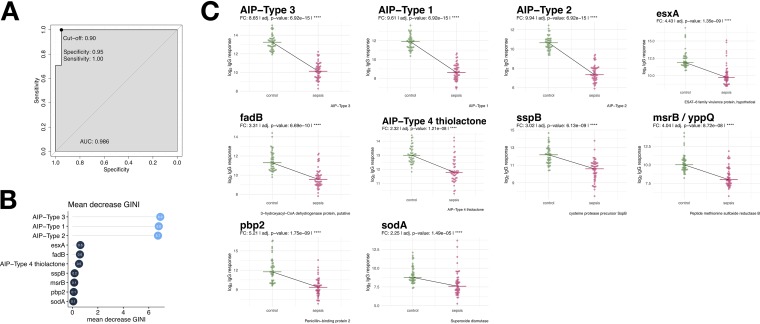
Random forest predictions. Random forest prediction was carried out using the caret package (75). The data were split into 50% test and 50% training data. The model training was performed using the “repeatedcv” with 15 resampling iterations and 30 repeats. (A) ROC curve of the test data. (B) Top 10 antigens with their corresponding mean decrease of GINI, which is a measure of feature relevance. The higher the mean decrease in the importance of GINI is, the better the value is for the positive prediction. (C) Bee-swarm plots of top 10 proteins from random forest prediction with Wilcoxon test *P* values and fold changes.

Considering the individual S. aureus antigens, IgG binding to 48 of the 143 antigens was significantly lower in SABSI than in controls, whereas only Ecb (SAOUHSC_01110), a fibrinogen-binding protein-like protein, elicited a higher IgG binding in SABSI patients (fold change of >2 and a *P* value of <0.05). IgG binding to the remaining 95 S. aureus antigens did not differ significantly between SABSI patients and controls. We further performed a random forest prediction in order to identity the antigens with the highest discriminatory power. Figure 6A provides a receiver operating characteristic (ROC) curve based on the top 10 antigens and revealed a specificity of 0.95 and a sensitivity of 1. These antigens correspond to the GINI (Gini coefficient) (Fig. 6B) where GINI is a measure of feature relevance. The highest specificity was observed for the autoinducing peptides (AIP1 to -3) followed by EsxA and FadB. Figure 6B depicts IgG binding to the 10 S. aureus antigens that exhibited the biggest differences (FC > 2.0 to 10) between SABSI and controls. Four of the illustrated antigens are the AIPs, and the remaining proteins are EsxA, FadB, SspB, MsrB, Pbp2, and SodA. PCA of the corresponding S. aureus-specific serum IgG binding titers clearly separated SABSI patients from controls (Fig. 5B).

## DISCUSSION

### General findings.

As the SABSI patients (*n* = 49) and control group patients (*n* = 43) were matched for age, gender, diabetes, and S. aureus nasal carriage status, the distributions were similar between the two groups. The median age was 67 years and 68 years, with a male predominance of 67% and 63%, respectively. Older age and male gender are known predisposing factors for SABSI (26). Diabetes is another predisposing factor for infections including SABSI, and the presence of diabetes has been associated with significantly higher mortality in patients with S. aureus bacteremia and infective endocarditis (27). In our study, 27% of the SABSI patients and 14% of the control patients had diabetes. We did not identify an increase in the 30-day all-cause mortality in SABSI patients with diabetes compared to SABSI patients without diabetes (14% versus 15%, respectively).

S. aureus nasal carriage occurs generally in ∼30% of the adult population (28), corresponding well with our findings of nasal carriage in 31% of SABSI patients and 28% of controls. Although being clustered to 15 CCs, 65% of the SABSI isolates clustered to four (CC45, CC15, CC5, and CC30) of the 10 most common human S. aureus CCs (29), and all but one SABSI patient (14/15) had isolates clustering to the same CC in both blood culture and nasal isolates, suggesting endogenous infection. Most bloodstream infections (∼80%) in nasal carriers are endogenous, meaning that both colonizing and infection S. aureus isolates have the same genotype (30, 31). The S. aureus nasal isolates of the control group clustered to seven CCs, with the most frequent being CC45 (3/9). However, DNA microarray analysis was not performed on three isolates. Being in an area with low MRSA incidence (32), it is not surprising that only two patients (4%) were infected with MRSA. Neither of these two patients was an S. aureus nasal carrier.

The central issue in SABSI is the dysregulation of innate and adaptive immunity. This was confirmed in our study, which showed (i) an acute-phase response and (ii) lower basal specific IgG in SABSI. DIA-MS analysis quantified 386 serum proteins with more than 2 unique peptides, including 9 that were significantly higher and 7 that were lower in SABSI than in the controls (Table 2). The majority of the upregulated proteins belonged to the acute-phase response signaling pathway, with the highest increases observed in SAA1, SAA2, and CRP, and the majority of the downregulated proteins were apolipoproteins.

Immunoproteomics revealed that at disease onset SABSI patients had lower S. aureus specific serum IgG than controls, which corroborates earlier data and makes a case for protection from SABSI by adaptive immunity. The IgG response to 48 out of 142 tested S. aureus antigens was significantly lower in SABSI patients than in controls, with IgG binding to AIPs showing the most dramatic decrease with the best predictive value for SABSI.

### DIA-MS serum proteomics.

Upregulation of SAAs and downregulation of apolipoprotein A proteins (APOAs) were expected, as they are components of high-density lipoproteins (HDLs). Circulating lipoproteins, especially HDLs, are able to bind lipopolysaccharides (LPSs) from Gram-negative bacteria and lipoteichoic acids from Gram-positive bacteria (like S. aureus) (33, 34). During infection, the major components of HDLs are altered. This alteration includes a decrease in HDL cholesterol (HDL-C), phospholipids, and APOA1 and an increase of SAA. APOA1 can be replaced by SAA during the acute-phase response, and SAA can influence HDL-mediated cholesterol metabolism through inhibiting selective cholesterol uptake, contributing to decreased HDL-C levels. Low levels of APOA1 have been associated with poor prognosis in infective endocarditis (35) and are independently related to 30-day mortality in sepsis (36). The downregulated protein APOB is known to sequester AIP1 of S. aureus. This inhibits the *agr* signaling pathway and limits invasive infection due to a decrease in the production of virulence factors (37). APOM was also downregulated in the SABSI patients. Plasma APOM has previously been suggested to be a biomarker for sepsis. Kumaraswamy and colleagues (38) demonstrated a correlation between decrease in APOM and severity of sepsis. APOM is a carrier for barrier-protective sphingosine-1-phosphate (S1P), which is important for vascular barrier protection. A decrease in APOM could contribute to the increased vascular leakage observed in sepsis and systemic inflammatory response syndromes (38).

CRP is one of the most-studied biomarkers in patients with bloodstream infections (39). An increase in CRP can be measured after only 8 to 12 h of acute onset of disease. It is commonly used in combination with procalcitonin (PCT). CRP and PCT have been reported to be highly associated with infection likelihood, sepsis severity, and septicemia, but due to low specificity, they should not be used as the only criterion when diagnosing sepsis (40). The median CRP was 195 mg/liter in the SABSI patients versus 3 mg/liter in the control group, normal range being <5 mg/liter. PCT was not part of our hospital’s diagnostic portfolio at the time of inclusion, and the concentration in serum would be too low to detect by proteomic analyses. One reason is that the protein is very short with a low number of tryptic peptides. So far, only one peptide could be identified in all plasma studies collected in the world peptide atlas, and the status of identification is still weak (https://db.systemsbiology.net/sbeams/cgi/PeptideAtlas/Search?action=GO&search_key=P01258).

In addition to SAAs and CRP, which were strongly increased by factors of between 45.9 and 99.3, increases were also observed in the serum concentrations of ceruloplasmin, haptoglobin, A1AG1, A1AT, VWF, and CD14 (upregulation of 1.5- to 3.3-fold, Table 2). Ceruloplasmin and haptoglobin have been reported to inhibit microbial iron uptake. As a ferroxidase, ceruloplasmin helps to mobilize iron from tissue and could therefore help starve invading pathogens of their essential iron nutrients. Bacteria, including S. aureus, use haptoglobin as iron source by binding hemoglobin-haptoglobin (Hb-Hp) complexes and extracting iron. However, because of their polymeric structure, Hp2-1 and Hp2-2 can induce agglutination of bacteria. Kelly and coworkers (41) reported an association of elevated haptoglobin plasma levels in critically ill patients with sepsis with a decreased risk of in-hospital mortality.

A1AG1, α1-acid glycoprotein (AGP), is one of the major acute-phase proteins. It increases in serum in response to infection and has been extensively studied mainly due to its drug binding behavior (42). It has also been suggested that AGP may bind toxic molecules produced by microorganisms, such as toxic lectins and bacterial lipopolysaccharides, thereby providing general protection against septic shock. In a single-center study from Brazil (43), the authors reported that an AGP level of ≤120 mg/dl was significantly associated with 96-h mortality in patients with severe sepsis.

A1AT, α1-antitrypsin (AAT), is also an acute-phase protein and is a member of the serine protease inhibitor (SERPIN) supergene family. Matamala and coworkers reported upregulation of full-length AAT protein and also expression of short transcripts of the SERPINA1 gene caused by human blood neutrophils in response to lipopolysaccharide (44). It has also been reported that a specific C-terminal fragment of AAT expresses an immunomodulatory function, particularly on human neutrophils during severe sepsis (45). The effects of AAT on neutrophil function might depend on the molecular form and binding partner of AAT (46).

von Willebrand factor is a blood glycoprotein involved in hemostasis, important in platelet adhesion to wound sites. Higher levels in severe sepsis or septic shock than in healthy controls have been observed previously, but no discriminating values regarding disease severity and organ involvement or prognostic value regarding outcome could be identified (47).

The last upregulated protein in our study was CD14. It is a glycoprotein on membrane surfaces of monocytes/macrophages and acts as a receptor for complexes of lipopolysaccharides (LPSs) and LPS binding protein (LBP). Although LPSs are considered the main ligand, CD14 also recognizes other pathogen-associated molecular patterns such as lipoteichoic acid. Gram-positive bacteria like S. aureus contain large amounts of peptidoglycan and lipoteichoic acid. Skjeflo and coworkers (48) demonstrated that simultaneous upstream inhibition of complement and CD14 efficiently attenuated S. aureus-induced inflammation in a human whole-blood model, suggesting that double blockade might be a promising treatment of sepsis.

In addition to apolipoproteins, kallistin was downregulated in our SABSI patients. Kallistin is a negative acute-phase protein, and kallistin levels have been reported to markedly diminish in patients with sepsis in addition to a long list of other types of diseases (49). It has been suggested to serve as a new biomarker for prediction of outcome (49).

### Immunoproteomics.

S. aureus expresses an arsenal of virulence factors contributing to pathogenesis, many of which are immunogenic. At diagnosis, the SABSI patients had significantly lower specific anti-S. aureus antibody titers than the control patients, while total serum IgG concentrations did not differ between the groups. In this study, care was taken to test SABSI patients in the early stages of their disease to assess the baseline levels of their anti-S. aureus antibody response before the onset of an immune response to the infection. The 10 anti-S. aureus IgG specificities showing the best random forest discrimination between SABSI patients and controls were 4 autoinducing peptides (AIP1 to -4), EsxA, FadB, SspB, MsrB, Pbp2, and SodA, all of which had significantly lower concentrations in all the SABSI patients.

Regulation of S. aureus virulence factors is mainly controlled by the accessory gene regulator (*agr*) quorum-sensing system, a cell-to-cell chemical communication system. The *agr* system responds to the extracellular concentration of the autoinducing peptides (AIPs). When AIPs accumulate to a critical concentration, the *agr* system upregulates the secretion of virulence factors and downregulates cell surface adhesion factors (50). The low IgG binding to AIPs 1 to 4 observed in SABSI patients in this study suggests a poor response to S. aureus virulence factors. It cannot be excluded, however, that antibodies were adsorbed by bacterial antigens. The decrease of APOB in SABSI would also be expected to increase the levels of AIPs during the first phase of S. aureus infection.

EsxA is a part of a specialized secretion pathway in S. aureus that contributes to the pathogenesis of blood-borne infections (51) and has been reported to interfere with host cell apoptotic pathways (52), whereas FadB is involved in the aerobic and anaerobic degradation of long-chain fatty acids via the beta-oxidation cycle (https://www.uniprot.org/uniprot/P21177) (53).

Staphopain B (SspB) is a cysteine protease secreted by S. aureus. Smagur and coworkers (54) described a mechanism involving SspB, which, in the absence of human serum, provoked necrotic and apoptosis-like forms of death in human neutrophils and monocytes. They concluded that SspB, particularly in the presence of staphylococcal protein A, could reduce the number of functional phagocytes at the infection sites and thereby facilitate colonization and spread of S. aureus. Ohbayashi and coworkers (55) reported that secreted staphopains can degrade collagen and fibrinogen, mechanisms that may cause tissue destruction and induce bleeding.

MsrB is an enzyme known to protect bacteria from oxidative stress and is a virulence factor. These enzymes are overproduced in response to cell wall-active antibiotics and are considered important in cell wall stress stimulus in S. aureus (56).

S. aureus has a highly cross-linked peptidoglycan layer that is an essential component of the cell envelope and necessary for maintaining bacterial shape, rigidity, and survival. Penicillin-binding proteins (PBPs), including PBP2, are transpeptidases that catalyze these cross-link formations, and this activity is the target of β-lactam and glycopeptide antibiotics (57). PBP2a, a variant of PBP2, is found in methicillin-resistant S. aureus (MRSA). PBP2a is encoded by the *mecA* gene and is required for the expression of high-level β-lactam resistance (58). Only two SABSI patients had MRSA in our study.

Superoxide dismutase (SodA) destroys superoxide anion radicals that are normally produced within the cells and are toxic. It may play a role in maintaining cell viability, especially in the exponential growth phase, and has a role in resisting external superoxide stress (59, 60).

There are reports on signatures of anti-S. aureus IgG associated with SABSI. Serum IgG binding to 8 conserved S. aureus proteins has been reported to enable stratification of patients with high and low risk of sepsis early in the course of S. aureus bacteremia (5). Lower levels of antibody to S. aureus exotoxins have also been described as associated with sepsis in hospitalized adults with invasive S. aureus infections (20). Our study corroborates and extends these results. Low titers of anti-S. aureus antibodies are associated with bloodstream infection and/or a severe disease course. However, in terms of the S. aureus antigens, our study revealed different and new anti-S. aureus IgG specificities associated with early SABSI. The main reason is probably a difference in the antigen test panels. Nine of the 10 antigens with the best discriminatory power in this study (EsxA, MsrB, FadB, SodA, and AIPs triggered by accessory gene regulator) were exclusive for our panel. They were included on the basis of *in vivo* proteomics experiments, because they were most strongly upregulated during S. aureus infection of cell lines or mice (8 to 32 h postinfection) (15, 61). This highlights the power of *in vivo* proteomics for specific antigen selection, which can be the key for the discovery of protective antibody targets using immunoproteomics.

To summarize briefly, we have combined DIA proteomics and immunoproteomics to work out specific markers for SABSI. The results showed an increase of acute-phase proteins associated with lowered lipoproteins, which can be regarded as a marker signature for sepsis. However, these may not necessarily be specific for SABSI as, e.g., CRP is an acute inflammatory protein that increases at sites of both infection and inflammation (62). Furthermore, our data made a strong case that a weak S. aureus-specific IgG response increased the risk of developing SABSI. They identify an antibody marker signature that may guide treatment decisions in the early phase of an S. aureus infection and may even indicate the causative agent. Finally, our study demonstrated that *in vivo* proteomics is an excellent tool to identify new antigen targets for active or passive vaccination.

This study has limitations. The numbers of SABSI patients and controls were relatively low and need to be extended in future validation studies (cases of >50 per group). The study was carried out in a specific region in Norway; hence, its results are not necessarily transferable to other regions in the world where other S. aureus strains are responsible for SABSI, which could influence the antibody responses to the antigenic marker panel. Furthermore, sepsis is often a multibacterial infection, and with respect to this, infection-related antigens from other pathogens should also be considered in the future. We also cannot exclude adsorption of IgGs by bacterial antigens.

### Conclusion.

DIA-MS proteomic analyses showed an acute-phase response in SABSI patients and evidence that the lipoproteins are downregulated. Using immunoproteomics, stratification of patients appears to be achievable, since patients at high risk of developing S. aureus infection can be identified by virtue of their low titers of preexisting anti-S. aureus antibodies. This strengthens the notion that a robust immune memory for S. aureus protects against infections with the pathogen.

## MATERIALS AND METHODS

### Study design and setting.

This prospective cohort study was performed at Akershus University Hospital (Ahus), Lørenskog, Norway. Ahus is the largest acute-care hospital in Norway with 650 beds and a catchment area of ∼500,000 inhabitants, corresponding to almost 10% of the Norwegian population. The study was approved by the South-Eastern Norway Regional Health Authority (2009/2149) and the local data protection officer, and all patients and controls gave their informed, written consent. To ensure adequate test power, the number of samples was chosen after prior estimation in G*Power version 3.1 (62). We used a two-tailed Wilcoxon test and an effect size of 1.5, an alpha error probability of 0.05, a power of 0.95, and an allocation ratio of 1 to calculate the necessary sample size. The calculation estimated a sample size of 14 samples with an actual power of 0.96. We have chosen 49 sepsis samples and 43 control samples for our study.

### Patients.

All patients ≥18 years of age with an S. aureus-positive blood culture collected from August 2012 through November 2013 were eligible for inclusion. We excluded patients who (i) were not resident in Ahus’ catchment area, (ii) had evidence of coinfection with other pathogens, or (iii) lacked clinical signs or symptoms of SABSI in the absence of antibiotic treatment, since this suggested contamination of blood culture with S. aureus. Moreover, we included only patients who were immunocompetent (defined as not receiving immunosuppression therapy or steroids in the last 3 months) and free of cancer or the need for dialysis over the previous 3 months. Finally, only patients who presented early during their disease course were included. This was defined by an onset of symptoms of ≤4 days prior to the sampling time point of the positive blood culture.

### Controls.

The control group consisted of patients ≥18 years of age undergoing elective orthopedic surgery (total hip or knee joint replacement, lumbar surgery) at Ahus. These patients were recruited in two periods during March 2011 through June 2014. The control patients were healthy enough to be approved for elective orthopedic surgery and did not develop an S. aureus surgical site infection within 1 year after surgery. Controls were matched according to sex, age, nasal carriage, and diabetes mellitus status.

### Demographics and clinical data.

We collected clinical information including sex, age, and 30-day all-cause case fatality (defined as death within 30 days after positive blood culture). Severity of infection was categorized as sepsis, severe sepsis, or septic shock according to the criteria specified by Levy and coworkers (63), as this was the international standard used at the time of inclusion.

### Bacterial isolates.

Nasal swabs were collected at inclusion from both patients and controls. S. aureus was identified according to routine diagnostic protocols including use of matrix-assisted laser desorption ionization–time of flight (MALDI-TOF) MS (Bruker, Bremen, Germany). Methicillin resistance was tested with a cefoxitin disc (EUCAST). Staphylococcus protein A gene (*spa*) typing was performed on all S. aureus isolates according to the method of Fossum Moen and coworkers (64). Genotyping was performed using DNA microarray (Alere Technologies, Jena, Germany) as previously published (3, 65, 66) to assign the clinical isolates to S. aureus clonal complexes, determine the prevalence of PVL genes, and allow detection of genes associated with antibiotic resistance.

### Collection of sera.

Sera from patients with SABSI were obtained from diagnostic blood samples taken as close to the sampling time of the positive blood culture as possible (0 to 3 days). Sera from controls were collected in connection with standard examinations prior to elective surgery.

### Total IgG.

Total IgG in serum was measured by turbidimetry using Vitros Chemistry Products IgG reagents (catalog no. 680 1733) and Vitros diluent pack 2 (catalog no. 680 1753) on a Vitros 5.1 FS instrument according to the manufacturer’s instructions (Ortho Clinical Diagnostics, Raritan, NJ).

### Tryptic digestion and sample preparation for mass spectrometry.

Buffered by 20 mM ammonium bicarbonate, 4 μg of serum protein was, first, reduced in the presence of 2.5 mM dithiothreitol (DTT) and, second, alkylated using 10 mM 2-iodoacetamide (IAA). Finally, proteins were digested into peptides by 160 ng sequencing-grade modified trypsin (Promega GmbH, Mannheim, Germany) dissolved in 20 mM aqueous ammonium bicarbonate per sample overnight. The digestion was stopped with acetic acid in a final concentration of 1%. Tryptic peptides were purified using ZipTip μ-C18 pipette tips (Merck Millipore, Billerica, MA, USA).

### Mass spectrometric data-dependent acquisition (DDA).

MS analyses were performed with an online coupled UltiMate 3000 RSLC system (Thermo Fisher Scientific, Idstein, Germany) connected to a Q Exactive Orbitrap MS (Thermo Fisher Scientific Inc.). For liquid chromatography (LC) separation, peptides were enriched on an Acclaim PepMap 100, 100-μm by 2-cm, nanoViper C18, 5 μM, 100-Å precolumn (Thermo Fisher Scientific Inc.) and separated using an Accucore 150-C18 column with a column length of 25 cm or 50 cm (150 Å, 2.6 μm; Thermo Fisher Scientific Inc.) and a temperature of 40°C. For separation, a 120-min gradient was used with a solvent mixture of buffer A (5% acetonitrile [ACN] in water with 0.1% acetic acid) and increasing percentages of buffer B (ACN with 0.1% acetic acid): 2% for 10 min, 2 to 25% for 120 min, 25 to 40% for 5 min, 40 to 90% for 2 min, and 90% for 5 min. Peptides were eluted with a flow rate of 300 nl/min for the 25-cm column and 200 nl/min for the 50-cm column. Full-scan MS was carried out using a mass range of *m/z* 300 to 1,650. Data were acquired in a data-dependent strategy in profile mode with a resolution of 70,000 for MS at *m/z* 400 and 17,500 for MS/MS and a positive polarity. The method used allowed sequential isolation of the top 10 most intense ions for fragmentation using high-energy collisional dissociation (HCD) with dynamic exclusion for 30 s and disabled early expiration. An intensity threshold of 8.3e4 was applied with an isolation width of 3 *m/z*, normalized collision energy of 27.5 eV, and a starting mass of *m/z* 100. The charge state screening and monoisotopic precursor selection rejected +1 and +7, +8, and >+8 charged ions.

### Mass spectrometric data-independent acquisition (DIA-MS).

LC-MS/MS analysis was performed on an UltiMate 3000 RSLC (Dionex/Thermo Fisher Scientific, Idstein, Germany) coupled to a Q Exactive mass spectrometer (Thermo Fisher Scientific, Waltham, MA, USA). The peptide digest was enriched on a 2-cm by 100-μm Acclaim PepMap 100 trap column (100-Å pore size, 5-μm C18 particles; Thermo Fisher Scientific) and separated on a 50-cm by 75-μm Accucore 150-C18 analytical column (150-Å pore size, 2.6-μm particles; Thermo Fisher Scientific) with a flow rate of 200 nl/min or a 25-cm by 75-μm Accucore 150-C18 analytical column (150-Å pore size, 2.6-μm particles; Thermo Fisher Scientific) with a flow rate of 300 nl/min at a constant temperature of 40°C. Reversed-phase chromatography was performed with a binary buffer system consisting of 0.1% acetic acid-5% ACN in water (buffer A) and 100% ACN in 0.1% acetic acid (buffer B). The peptides were separated by applying a linear gradient from 2% to 25% buffer B over a time of 120 min and 180 min for the 25-cm and the 50-cm analytical column, respectively. Eluting peptides were ionized using the chip-based TriVersa NanoMate ion source (Advion Biosciences, Norwich, United Kingdom). For the data-independent mode, the parameters from the work of Bruderer and coworkers (67) were used.

### Building a human plasma ion library for DIA-MS data analysis.

Ninety measurements of human plasma protein extracts (depleted for albumin, IgG, antitrypsin, IgA, transferrin, and haptoglobin using multiaffinity chromatography [MARS6-human; Agilent Technologies, Waldbronn, Germany] [68] and nondepleted) with spiked-in iRT (indexed retention time) peptides were searched against the human UniProt canonical database (status of June 2015). Nonhuman common contaminants listed in the Common Repository of Adventitious Proteins (cRAP), iRT peptides, and a sequence-reversed decoy counterpart were appended to the database. For the search, the parent mass error was set to ±30 ppm, and peptides were allowed to be semitryptic with up to two internal cleavage sites. The search parameters included a fixed modification of +57.021464 for carbamidomethylated cysteine and a variable modification of +15.9949 for oxidized methionines. The individual Comet search iProphet results were converted to a blib file using ProteoWizard 3.0.7364 (69) (cutoff score = 0.9). The nonredundant blib files were used to generate a library using the bioconductor package specL (1.5.2) (69) (parameter: maximum MZ (*m/z*, mass to charge) error, 0.01 Da; TopN, 10; fragment ion *m/z* range, 300 to 2,000; fragment ion type, b,y]. The final library was filtered for 6 to 10 transitions and the lowest fragment mass error.

### DIA-MS data analysis of serum.

The DIA-MS data analysis was performed using Spectronaut (v9.0.11240.15) using a dynamic XIC (extracted ion chromatogram) extraction window with a correction factor of 1 and dynamic score refinement for identification and including the interference correction for the quantification, as described previously (15). The area under the curve for each ion was reported.

### Immunoproteome analysis.

The analysis of the immune response to S. aureus antigens was performed using the Luminex xMAP technology with 130 S. aureus proteins (142 antigens) as described previously (5). The values were calculated as described previously (5) with the change that a linear regression was used for antigens whose 1:50 dilution was still in the linear range. The response value in that case is calculated by multiplying the dilution (0.02) with the intensity at that dilution which results in a very low response value.

### Data analyses and statistics.

The DIA-MS data were filtered for a *q* value of <0.001, nonunique peptides were removed, and subsequently the quantitative data were globally normalized using the median. Statistical testing was performed protein-wise with a Wilcoxon rank sum test against an absolute fold change of 1.5 using Benjamini and Hochberg’s multiple testing correction using R (v3.1) (70), as described previously (15). The processed immune response data obtained from the immune proteome analysis were statistically tested with a Wilcoxon rank sum test between SABSI patients and control group using R (v3.1) (70) and the ggpubr package (version 0.2) (71). The *P* value was adjusted using Benjamini and Hochberg’s multiple testing correction. The principal-component analysis (PCA) was performed in R (R version 3.5) (70) using the FactoMineR package (version 1.36) (72). To unify variances, scaling of data was done by dividing the (centered) columns of the data by their standard deviations. The Hierarchical Clustering on Principal Components (HCPC) performs an agglomerative hierarchical clustering on results from a factor analysis like PCA (metric, Euclidean; linkage, average). The cutting of the tree into clusters was done with three clusters. PCAs were plotted using the Factoextra package (73) (version 1.0.5) and ggplot2 package (74) (version 3.1.0). The random forest prediction of immunoproteomics data was performed by splitting the data into a test and a training set (percentage of data for training/test = 50% each). The random forest prediction was carried out using the caret package (version 6.0-80) in R (75). The training control was done with 30 separate 15-fold cross-validations as resampling scheme. A ROC curve was plotted based on the prediction using the trained model and the test data subset.

All other clinically related statistical tests were done using the IBM SPSS Statistics software version 23 (SPSS Inc., Chicago, IL, USA).

10.1128/mSystems.00632-19.4 TABLE S3 DIA-MS statistics. The column heads are defined as follows: Protein_ID, UniProt protein identifier; median_over_assay_ratios, median over protein assay ratio matrix per protein; fold_change, fold change; ratio_meta, how ratios were calculated; IQR_of_assay_ratios, interquartile range (IQR) over assay ratio matrix per protein; test_method, statistical test used; p_value, *P* value; ratios_tested_against_location, location tested against (one-sided test); Alternative, alternative (one-sided test); confidence_interval_95percent_lower, lower 95% confidence interval (only if alternative is greater); confidence_interval_95percent_upper, upper 95% confidence interval (only if alternative is less); (pseudo)median, estimate from the test; p_value_BH_adjusted, Benjamini-Hochberg (BH) adjusted *P* value. Download Table S3, XLSX file, 0.04 MB. Copyright © 2020 Michalik et al. 2020 Michalik et al. This content is distributed under the terms of the Creative Commons Attribution 4.0 International license.

10.1128/mSystems.00632-19.5 TABLE S4 Immunoproteomic statistics. The column heads are defined as follows: Antigen, antigen; gene_symbol, antigen gene symbol; Description, antigen protein description; ratio_meta, how ratios were calculated; p_value, *P* value; p_value_BH_adjusted, Benjamini-Hochberg (BH) adjusted *P* value; test_method, statistical test used; ratio_control_vs_sepsis, ratio; median_response_control, median over response of control subjects; median_response_sepsis, median over response of sepsis patients; fold_change, fold change. Download Table S4, XLSX file, 0.03 MB. Copyright © 2020 Michalik et al. 2020 Michalik et al. This content is distributed under the terms of the Creative Commons Attribution 4.0 International license.
