# Supplementary figures and images for: Early-Stage Staphylococcus aureus Bloodstream Infection Causes Changes in the Concentrations of Lipoproteins and Acute-Phase Proteins and Is Associated with Low Antibody Titers against Bacterial Virulence Factors
Source: mSystems. 2020 Jan 21;5(1):e00632-19. doi: 10.1128/mSystems.00632-19 (PMC6977072; doi:10.1128/mSystems.00632-19)

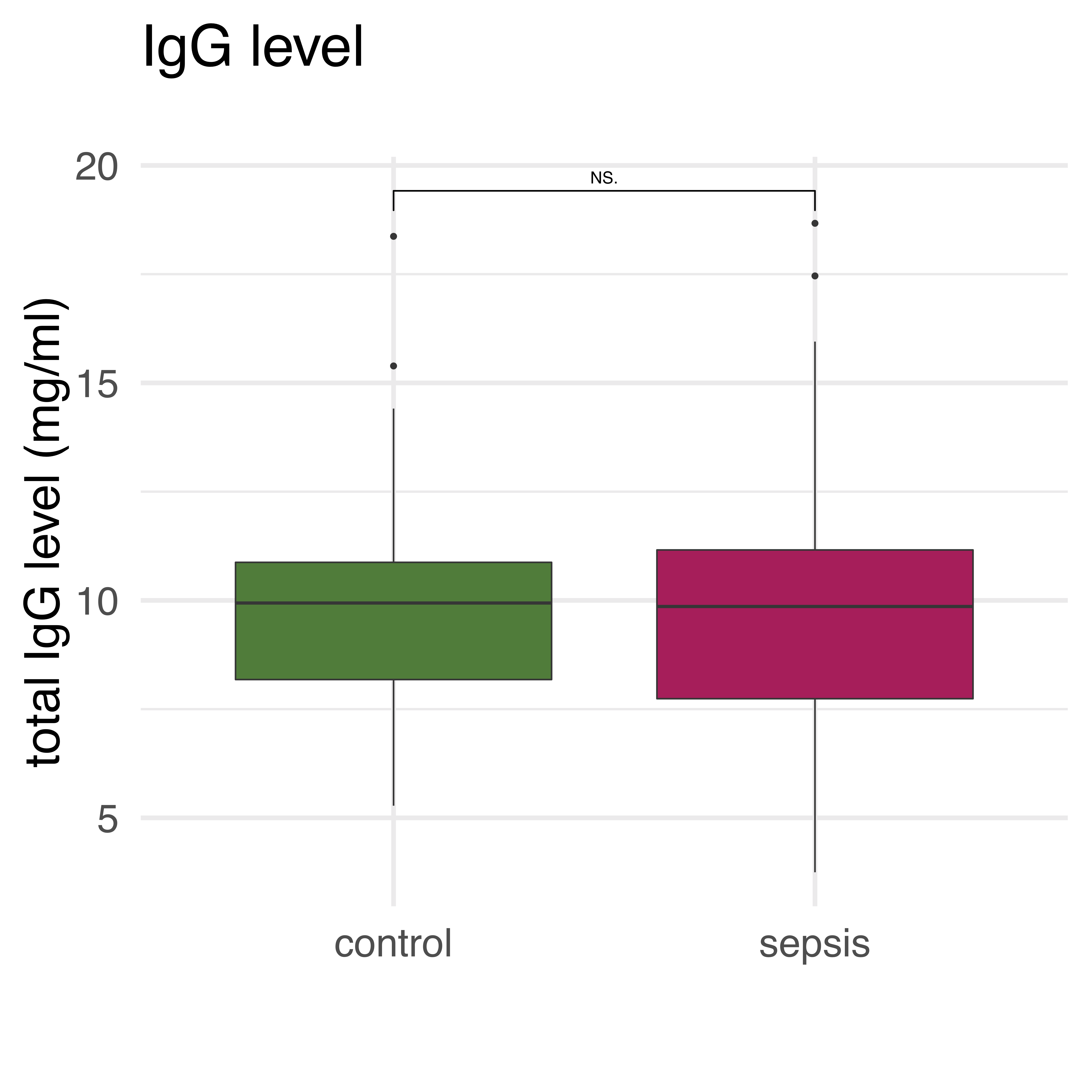

Supplement: FIG S1 [file mSystems.00632-19-sf001.tif]
